# Carbapenem-resistant *Enterobacterales* peri-rectal colonization prevalence on admission to two intensive care units in an academic hospital in India

**DOI:** 10.1017/ash.2025.10036

**Published:** 2025-05-30

**Authors:** Armaghan-e-Rehman Mansoor, Fabia Edathadathil, Devendhu Suresh, Yathu Krishna, Anu George, Jacaranda van Rheenen, Ige A George, Jennie H Kwon, Emily E Petersen, Matthew Westercamp, Anil Kumar, Sudheer O Vayoth, Margaret A Olsen, Surbhi Leekha, Sanjeev K Singh, David K Warren, Sumanth Gandra

**Affiliations:** 1 Division of Infectious Diseases, Department of Internal Medicine, University of Kentucky, Kentucky, USA; 2 Amrita Institute of Medical Sciences, Kochi, KL, India; 3 Division of Infectious Diseases, Department of Medicine, Washington University School of Medicine in St. Louis, St. Louis, USA; 4 Division of Healthcare Quality Promotion, Centers for Disease Control and Prevention, Atlanta, GA, USA; 5 Department of Epidemiology and Public Health, University of Maryland School of Medicine, Baltimore, USA

## Abstract

This study from a South Indian tertiary care hospital found a 41% peri-rectal Carbapenem-resistant *Enterobacterales* colonization prevalence at intensive care unit admission, with New Delhi metallo-β-lactamase as the predominant carbapenemase. It underscores the need for contextually appropriate, cost-effective infection prevention strategies to mitigate the spread of resistant organisms in Indian healthcare settings.

## Introduction

Carbapenem-resistant *Enterobacterales* (CRE) infections are endemic in Indian healthcare facilities and are the leading cause of hospital-acquired infections^1^. Despite this high prevalence, few studies have examined the CRE colonization status on admission to adult intensive care units (ICUs) in India^2–7^. Examining CRE peri-rectal colonization status can aid in informing infection prevention measures, and assess subsequent clinical infection and transmission risk. In this study, we investigated the prevalence of CRE peri-rectal colonization and carbapenemase type among patients admitted to two ICUs at a tertiary care academic hospital in India.

## Methods

The study was conducted at a 1250-bed private academic tertiary care hospital in South India. This study was approved by the Human Research Protection Office at Washington University School of Medicine, the study hospital ethics committee, and the Indian Health Ministry’s Screening Committee. We enrolled patients admitted to two adult ICUs: the medical ICU (MICU) and the gastrointestinal surgical ICU (SICU). The MICU is organized into eight cubicles, each with four beds, while the SICU consists of a single hall with 11 beds arranged side by side.

From December 16, 2022, to April 30, 2023, all patients admitted to the MICU and SICU with an anticipated stay of more than 24 hours as assessed by physicians were approached for enrollment upon obtaining informed consent. Peri-rectal swabs (HiMedia Laboratory Pvt. Ltd., Mumbai, India) obtained within 48 hours of admission were used to assess CRE colonization status at the time of ICU admission.

The collected swabs were plated on mSuperCARBA selective agar medium (CHROMagar, Paris, France) within 24 hours of collection and incubated under aerobic conditions at 35–37°C for 18–24 hours to isolate CRE. Quality control for the selective media was performed as per manufacturer’s instructions. Identification and susceptibility testing of isolates presumed to be CRE were performed using the VITEK2 system (bioMérieux; Marcy-l’Étoile, France). The molecular mechanism of carbapenemase-mediated resistance was determined using the Xpert Carba-R assay (Cepheid, Sunnyvale, CA).

Information on select risk factors for CRE colonization^8^, including hospitalization and ICU stay within the last year, transfer from an outside hospital, and long-term hemodialysis status was collected by interviewing patient and or caregiver. Statistical analysis included Chi-Square tests for categorical variables and either Student’s t-test or Mann—Whitney U test for continuous variables, with a significance threshold of *P* < 0.05. Missing data on variables were excluded from the denominators. All analyses were conducted using SPSS Statistics 27 software (IBM, Armonk, NY).

## Results

**Figure 1.**
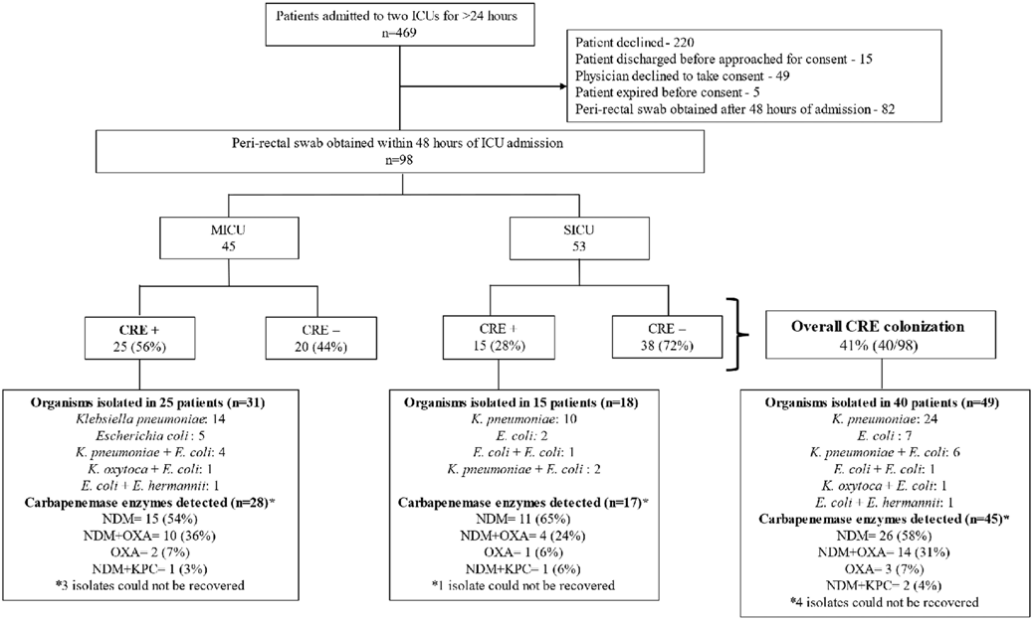
Legend—Enrollment, carbapenem-resistant *Enterobacterales* colonization prevalence, organism distribution and carbapenemase enzyme detected in the medical and surgical intensive care units (ICU) of a tertiary care hospital, Kerala, India, December 2022-April 2023.

Out of 469 patients admitted to the two ICUs for more than 24 hours, peri-rectal swabs were collected within 48 hours of ICU admission from 98 patients (21%): 45 in the MICU and 53 from the SICU (Figure 1). The median hospital stay prior to ICU admission was shorter for patients entering the MICU (0 days, IQR 0-1) compared to those in the SICU (1 day, IQR 1-2; *P* = 0.01) (Table 1).

**Table 1.** Demographics and characteristics of enrolled patients in the two intensive care units in a tertiary care hospital, Kerala, India, December 2022–April 2023

	Total n (%) N = 98	MICU n (%) N = 45	SICU n (%) N = 53	P-value
Male	65 (66%)	35 (80%)	30 (57%)	0.030
Median age in years (IQR)	58 (46–68)	65 (54–69)	55 (35–66)	0.010
Median LOS in the study hospital prior to ICU admission (days) (IQR)	1 (0-2)	0 (0-1)	1 (1-2)	0.010
Prior hospitalization in last one year^	49/73 (67%)	29/35 (83%)	20/38 (53%)	0.007
Prior ICU stay in last one year^	22/62 (35%)	11/29 (38%)	11/33 (33%)	0.710
Long-term hemodialysis^	12/91 (13%)	10/41 (24%)	2/50 (4%)	0.005
Outside hospital stay prior to ICU admission^	26/76 (34%)	13/36 (36%)	13/40 (33%)	0.740

^ denominators are different due to missing data; IQR = interquartile range; LOS = length of stay; ICU = intensive care unit.

Patients admitted to the MICU were significantly more likely to have been hospitalized in the last year (29/35; 83% [95% confidence interval [CI]: 71% – 95%]) compared to those in the SICU (20/38; 53% [95% CI: 37% – 69%]; *P* = 0.007). In addition, a greater proportion of MICU patients required long-term dialysis (10/41; 24% [95% CI: 11% – 37%]) compared to SICU patients (2/50; 4% [95% CI: -1% – 9%]; P = 0.005) (Table 1).

CRE peri-rectal colonization was detected in 41% (95% CI: 31% – 51%) of patients (40/98) within 48 hours of ICU admission, with higher rates among patients admitted to the MICU (25/45; 56% [95% CI: 42% – 71%]) compared to the SICU (15/53; 28% [95% CI: 16% – 40%]; *P* = 0.007). Of the colonized patients, 49 CRE organisms were isolated, with nine patients having two different CRE organisms. *Klebsiella pneumoniae* was the most prevalent, accounting for 61% of isolates (30/49), followed by *Escherichia coli* at 29% (14/49) (Figure 1).

Molecular testing was performed on 45 CRE isolates, with four CRE isolates not recoverable (Figure 1). Among the tested isolates, the NDM enzyme was present in 58% (26/45). Additionally, NDM and OXA-48 were identified in 31% (14/45), OXA-48 alone in 7% (3/45), and NDM and KPC together in 4% (2/45).

## Discussion

The reported prevalence of CRE colonization at ICU admission in India has varied widely, ranging from 8% to 35% in studies conducted from 2011 to 2022^2–7^. The most recent study, conducted between 2019 and 2022 in a central Indian state, reported a prevalence of 13%^5^ while we observed a substantially higher prevalence of 41%. The higher prevalence in our study could be due to regional differences; however, two specific methodological differences are noteworthy. First, unlike previous studies, we adopted a more inclusive approach to defining our study population. We did not exclude patients based on transfers within or between facilities prior to enrollment, which could have resulted in a sample with greater CRE exposure. Second, we utilized a more sensitive selective chromogenic agar^9^, in contrast to the MacConkey agar impregnated with meropenem used by the majority of earlier studies. The use of chromogenic agar, also employed by the 2019 study by Mahapatra et al. in Eastern India, which reported a CRE prevalence of 35%^6^, aligns more closely with our findings and could explain the higher prevalence observed in our study. Lastly, swabs were collected between 24 and 48 hours after admission, making it possible that some CRE acquisition occurred in the ICU. Previous studies have rarely detailed the molecular mechanisms underlying CRE resistance at ICU admission. In our study, a substantial 93% of CRE isolates harbored the NDM enzyme, alone or in combination with OXA-48 or KPC, with OXA-48 detected in 38% of isolates. This aligns with the 2021 Indian National Surveillance Report^10^, which identified OXA-48 followed by NDM, as the most frequent enzymes in clinical CRE isolates, with variation across facilities where one of these enzymes was predominant. The high prevalence of NDM poses therapeutic challenges due to lack of effective and affordable antibiotics.

The notably higher prevalence of CRE colonization in the MICU, compared to the SICU, can be linked to the higher proportion of previous healthcare exposure among MICU patients and more complex underlying medical conditions. This highlights the need for targeted infection prevention strategies, particularly in settings with high patient turnover and complex care requirements. The burden of CRE colonization, coupled with the limited efficacy of existing treatments against NDM-producing organisms, underscores the need for enhanced prevention measures and the development of novel prevention strategies.

Our study’s limited enrollment (21% of admissions), influenced by cultural norms affecting the consent process and brief stays in the SICU, introduces uncertainty in the true baseline prevalence of CRE at ICU admission. Moreover, incomplete records restricted our ability to thoroughly assess prior healthcare and antibiotic exposures; therefore, we could not explore patient-level risk factors in greater detail.

In conclusion, peri-rectal CRE colonization among patients admitted to two ICUs in a South Indian hospital was 41%, with a significantly higher prevalence in the MICU when compared to SICU. Future research should examine pre-hospital exposures, particularly those preceding admission to tertiary care centers, and should focus on examining the risk factors associated with CRE acquisition during hospitalization and transmission dynamics of CRE within hospital settings in India to facilitate the development of contextually appropriate prevention strategies aimed at curtailing CRE spread.
